# Exploring metallic and plastic 3D printed photochemical reactors for customizing chemical synthesis

**DOI:** 10.1038/s41598-022-07583-9

**Published:** 2022-03-08

**Authors:** Evgeniy G. Gordeev, Kirill S. Erokhin, Andrey D. Kobelev, Julia V. Burykina, Pavel V. Novikov, Valentine P. Ananikov

**Affiliations:** 1 Zelinsky Institute of Organic Chemistry, Russian Academy of Sciences, Leninsky Prospect 47, Moscow, Russia 119991; 2 Lomonosov Moscow State University, Leninskie Gory GSP-1, 1-3, Moscow, Russia 119991

**Keywords:** Synthetic chemistry methodology, Process chemistry, Chemical engineering

## Abstract

Visible light photocatalysis is a rapidly developing branch of chemical synthesis with outstanding sustainable potential and improved reaction design. However, the challenge is that many particular chemical reactions may require dedicated tuned photoreactors to achieve maximal efficiency. This is a critical stumbling block unless the possibility for reactor design becomes available directly in the laboratories. In this work, customized laboratory photoreactors were developed with temperature stabilization and the ability to adapt different LED light sources of various wavelengths. We explore two important concepts for the design of photoreactors: reactors for performing multiple parallel experiments and reactors suitable for scale-up synthesis, allowing a rapid increase in the product amount. Reactors of the first type were efficiently made of metal using metal laser sintering, and reactors of the second type were successfully manufactured from plastic using fused filament fabrication. Practical evaluation has shown good accuracy of the temperature stabilization in the range typically required for organic synthesis for both types of reactors. Synthetic application of 3D printed reactors has shown good utility in test reactions—furan C–H arylation and thiol-yne coupling. The critical effect of temperature stabilization was established for the furan arylation reaction: heating of the reaction mixture may lead to the total vanishing of photochemical effect.

## Introduction

In recent decades, photocatalysis has become one of the most attractive areas of research in chemistry and material science^1–3^. An important advantage of light-mediated reactions concerns the implementation of the principles of “green chemistry” and sustainable development. In many cases, photocatalysis allows chemical transformations to be conducted under mild conditions, replacing traditional procedures that require much harsher experimental conditions or highly toxic catalysts containing expensive metals and metal complexes. Photocatalysis has been successfully applied for water splitting and hydrogen generation^4–6^, reduction of carbon monoxide and carbon dioxide^7–10^, wastewater detoxification and oxidative decomposition of organic pollutants^11,12^. The outstanding potential of photocatalysis has been explored in the field of organic synthesis with respect to the transformation of fine chemicals and the production of biologically active molecules and pharmacological substances^13–24^.

Classical photochemistry is equally important for synthetic transformations and has found numerous applications^25–28^. Representative examples include photochemical activation of cycloaddition reactions^29,30^, carbon skeleton re-arrangements^31,32^, addition to multiple bonds^33^, photoisomerization^34,35^ and many radical reactions^36,37^. Important practical applications of photochemistry include medicinal chemistry^38,39^, fine chemicals^40^, access to polycyclic compounds^41^, new materials^42^, biomass conversion^43^, organometallic chemistry^44^, among many others^45,46^.

The potential impact of photochemistry and photocatalysis on synthetic applications greatly depends on the availability of dedicated laboratory equipment^47,48^. For maximum efficiency, individual reactions require specially designed and tuned reactors. Researchers are trying to design such reactors, but a problem often arises here. The diversity of in-house photochemical setups that are frequently used for carrying out chemical transformations often makes it difficult to reproduce the high efficiencies and selectivity reported. Of particular importance is temperature stabilization during the photochemical process. Indeed, producing well-defined and reproducible photocatalytic experiments appears to be nearly impossible without temperature control.

Significant progress in this area may be achieved by the design and manufacturing of customized equipment by additive manufacturing (3D printing)^49,50^. The area of 3D printing has experienced tremendous growth, and it has already been widely incorporated in different fields of science and technology^51,52^. The progress of 3D printing has been stimulated by gradual improvements and cost reduction of the electronic components of 3D printers and the high efficiency of 3D modeling software. Of particular importance is the development of flexible and simple-to-use software (slicers) for the preparation of 3D models for additive manufacturing processes. Modern slicers allow the optimization of a wide range of parameters to obtain products of the highest quality using additive manufacturing. As a result, in recent years, low-cost and highly efficient photoreactors for a wide range of chemical processes have been actively developed using 3D printing^53–61^.

Regarding laboratory equipment, additive manufacturing has greater possibilities for the creation of customized reactors than conventional methods. 3D printing and microprinting are especially relevant for state-of-the-art chemical technologies^62–66^. The key advantage is the easy reproduction of 3D printed reactors once a relevant model is developed. Thus, 3D printed photoreactors retain the full ability of customized design and merge the possibilities for achieving high performance and reproducibility.

The most popular technologies for 3D printing with metals involve selective laser sintering (SLS) or melting (SLM), particularly direct metal laser sintering (DMLS). This technology is suitable for the manufacturing of small and middle-sized devices of virtually any complexity with high accuracy (including chemical reactors). Powder-based methods of 3D printing are compatible with a wide range of metal alloys and provide flexibility in choosing the most suitable material for the production of chemical reactors^67,68^.

However, the printing of metal parts is still not a very common method in chemical laboratory practice due to the very high price of the corresponding 3D printers and the complexity of the printing process. Therefore, despite the many advantages of metal products over plastic products (high thermal conductivity, strength and impermeability), 3D metal printing has not yet found widespread use in everyday laboratory practice. The most common, inexpensive and simple 3D printing method today is the fused filament fabrication (FFF) method, which makes it possible to manufacture products from a wide range of thermoplastic polymer materials, including those characterized by high chemical resistance^69^. The use of FFF printing does not require high qualifications or special engineering training. Therefore, FFF printing has found wide application for creating laboratory chemical reactors. In this work, both technologies (FFF and DMLS) were tested to create photoreactors with a different approach for the further use of reactors: metal reactors for parallel experiments and plastic reactors with the possibility of scaling the productivity of chemical synthesis.

In this work, two types of reactors have been developed to solve important problems in fine organic synthesis: optimization of the reaction conditions (type I) and scaling up the reaction to obtain large amounts of a product (type II) under the conditions of photochemical synthesis. The type I reactor allows many reactions to be carried out in parallel with a small amount of chemicals (typically, mg scale), and the type II reactor allows a single reaction to be carried out on a larger amount of chemicals (typically, grams scale).

## Results and discussion

**Figure 1:**
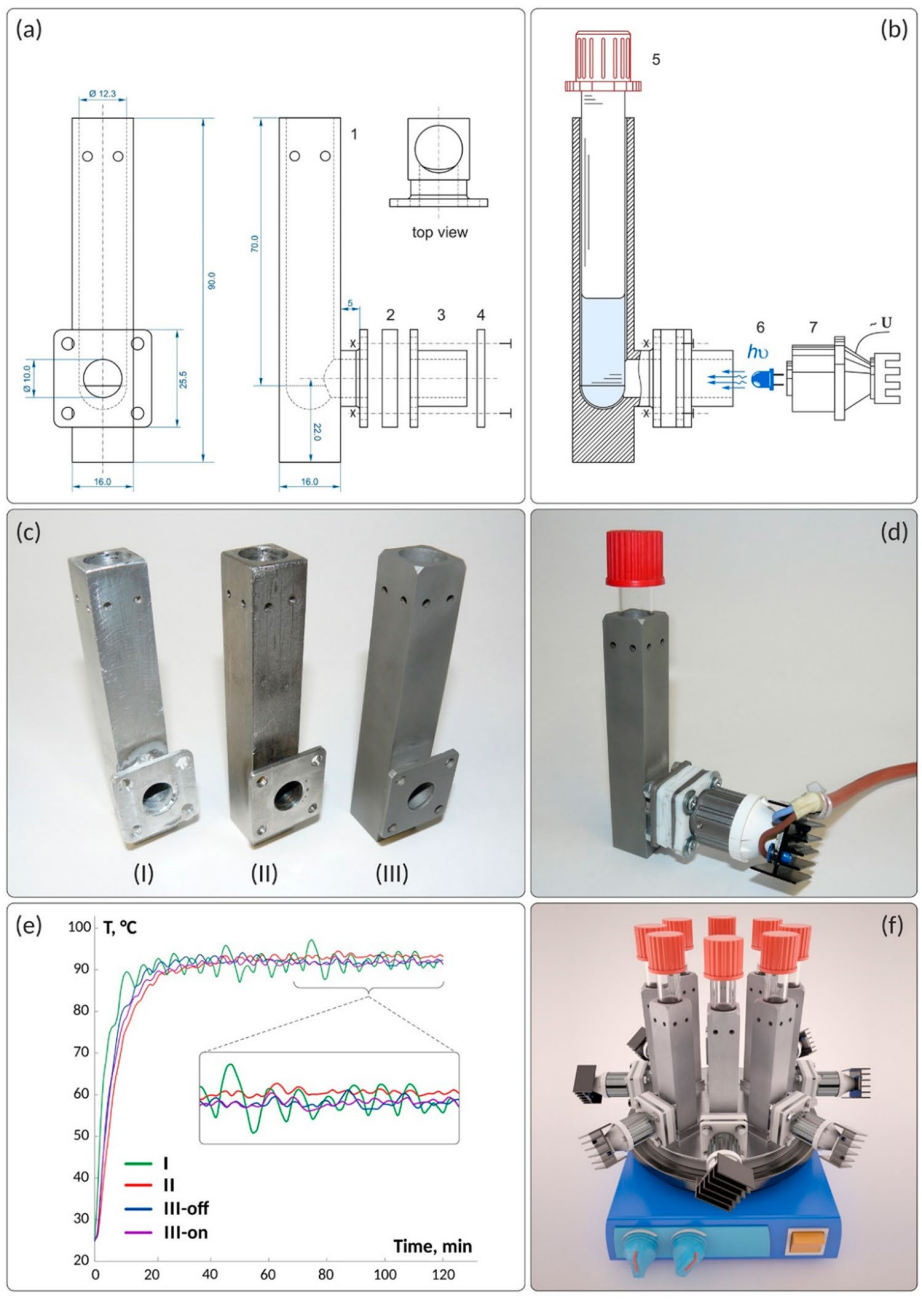
(**a**) Designed photoreactor with main dimensions shown in millimeters; (**b**) schematic representation of the assembling photoreactor with LED and test tube; (**c**) photos of the reactors made by a conventional cutting/milling/welding method from aluminum alloy (I) and stainless steel (II), as well as a reactor made of stainless steel by DMLS 3D printing (III); (**d**) photo of the assembled reactor with a test tube inside and a connected LED light source; (**e**) monitoring of temperature stability over a time period of 2 h in DMF solution at 90 °C for the following photoreactors: I—aluminum alloy and II—stainless steel reactors manufactured by conventional method; III-off and III-on—3D printed stainless-steel reactor with LED switched off and with LED switched on, respectively; (**f**) a 3D model showing several photoreactors for parallel operation under temperature control.

**Figure 2:**
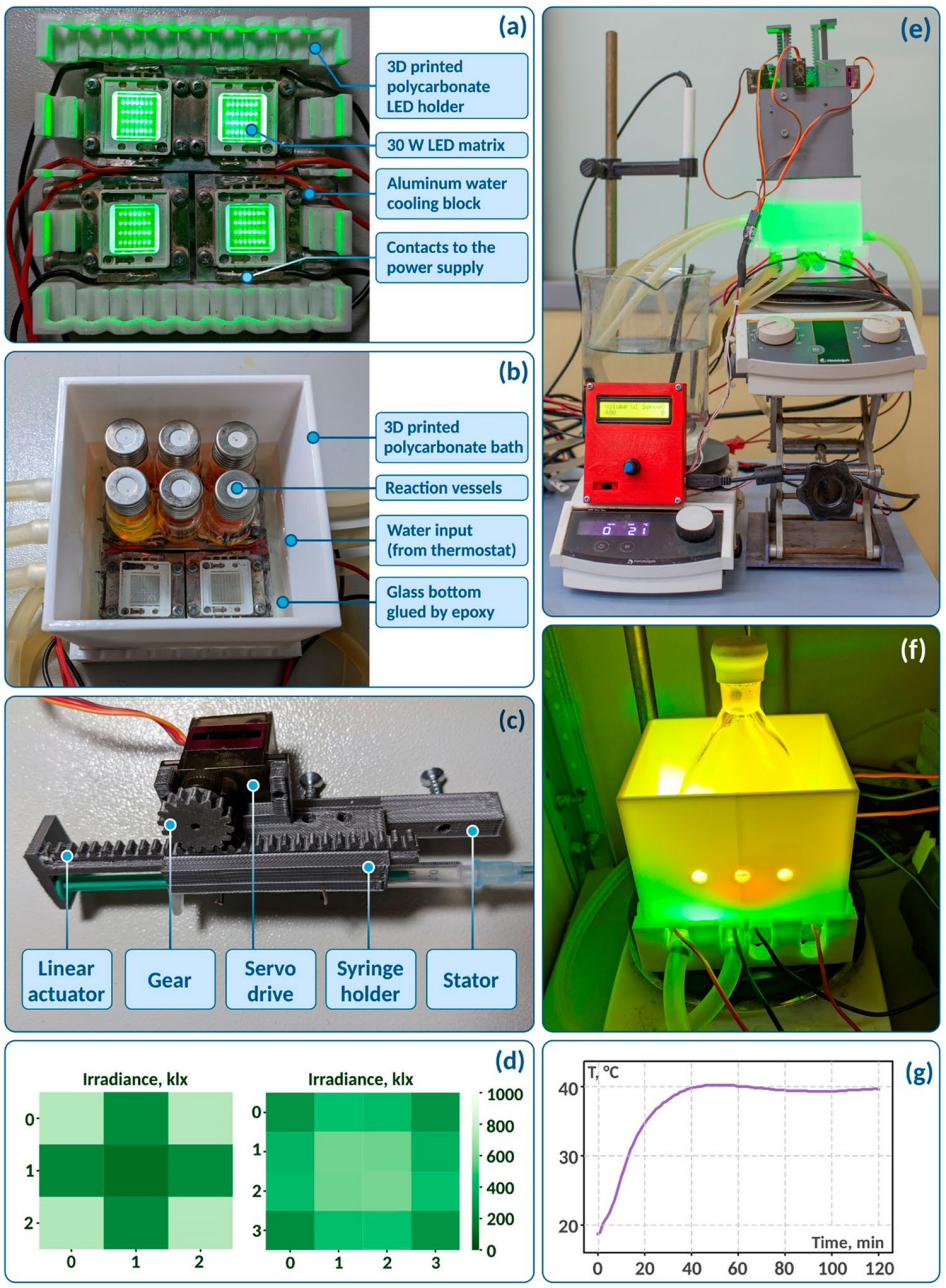
Custom build 3D printed photoreactor: (**a**) first layer is a matrix of four 30 W LEDs, cooled by water; (**b**) second layer is a full jacket with glass bottom; (**c**) third layer consists of four independent syringe pumps, made of 3D printed gears and controlling by servo motors and Arduino; (**d**) heatmaps of irradiance for possible vial positioning under LEDs measured with photodiode circuit; (**e**) completely assembled photoreactor; (**f**) gram-scale setup; (**g**) monitoring of temperature stability over a time period of 2 h at 40 °C. See Supplementary Figs. S3–S6 for dimensions of main parts of FFF photoreactor.

Optimization of reaction conditions and development of methodology is the first stage in organic synthesis. To address this aim, compact metal reactors have been developed, allowing the installation of several reactors to carry out many experiments at once to accelerate the optimization of organic synthesis conditions^70^. In the present work, we designed photochemical reactors that may be effectively manufactured using 3D printing technology (Figs. 1, 2).

The metal reactor (Fig. 1, Supplementary Fig. S1) was made of PH1 stainless steel and manufactured by DMLS using an EOSINT M280 sintering machine. The use of selective laser sintering technology in the present case resulted in the construction of a high-quality metallic reactor directly suitable for chemical applications.

The reactor center has a cylindrical main channel with a hemispherical bottom for the installation of standard screw-capped glass tubes (Fig. 1a,b). The bottom side of the reactor was provided with a side cylindrical channel crossing the main reactor channel to supply LED light. Commercially available light-emitting diodes can be easily installed for carrying out photochemical transformation, and reactors may be assembled in arrays for parallel experiments under the same temperature conditions (Fig. 1f). PTFE gaskets (2) and (4) were inserted for insulation of the LED from the reactor’s body (1) to prevent overheating (Fig. 1a). Various LEDs of different wavelengths (6) can be connected through a bayonet mount (3)–(7), making light source replacement within a few seconds possible.

For comparative study, we made two reactors using a conventional cutting/milling/welding process from aluminum alloy (I) and stainless steel (II) (Fig. 1c). A photochemical reactor made by 3D printing (III) possessed the same dimensions, while a considerably better dimension accuracy and visual quality of the metal surface may be noted which were achieved without additional post-processing. It should be noted that the usual methods of metalworking, of course, allow you to achieve high quality products, but this can require much more labor. All of the reactors were made according to our design experiment, and a tube (5) was inserted into the central channel to reach the hemispherical bottom of the reactor (Fig. 1b). A camera (7) containing a LED (6) was placed on the bayonet connector (3) and connected to the power supply, resulting in the fully assembled device (Fig. 1d).

A comparative analysis of temperature stabilization was carried out in manufactured reactors I, II and III (Fig. 1e). In the case of the 3D printed reactor, temperature stabilization was evaluated in the LED-on and LED-off modes (III-on and III-off, respectively). The experiment involved monitoring the solvent temperatures inside the test tubes, which were placed in the photoreactors for 2 h. The first benchmarking experiment was carried out in DMF at 90 °C. Less accurate temperature stabilization was observed in the aluminum reactor made by conventional technology, where the standard temperature deviation was approximately 2.0 °C (I, Fig. 1e). Good temperature stabilization with a standard temperature deviation of < 0.6 °C was observed in the stainless steel reactor made by conventional technology (II, Fig. 1e). Similar temperature stabilization was observed in the 3D printed reactor: in both LED-on and LED-off modes, the standard temperature deviations were < 0.6 °C (III-on and III-off, Fig. 1e). A second benchmarking experiment was carried out in water at 50 °C, where the same tendency was found (Supplementary Fig. S2). In reactor I, a standard temperature deviation of 1.5 °C was observed, whereas in reactors II, III-on and III-off, the standard deviations were < 0.5 °C. Thus, the 3D printed reactor made in the present study shows excellent temperature stabilization properties. The better performance of stainless steel compared to aluminum is also worth mentioning.

It should be noted that for photochemical processes, temperature is highly important but difficult to control; therefore, the possibility of installing compact metal reactors on one magnetic stirrer, providing the same heating and stirring, makes it possible to optimize the photochemical parameters, all other things being equal. This enables to better separate the effect of light exposure from the effect of temperature on the mechanism of a chemical reaction.

**Figure 3:**
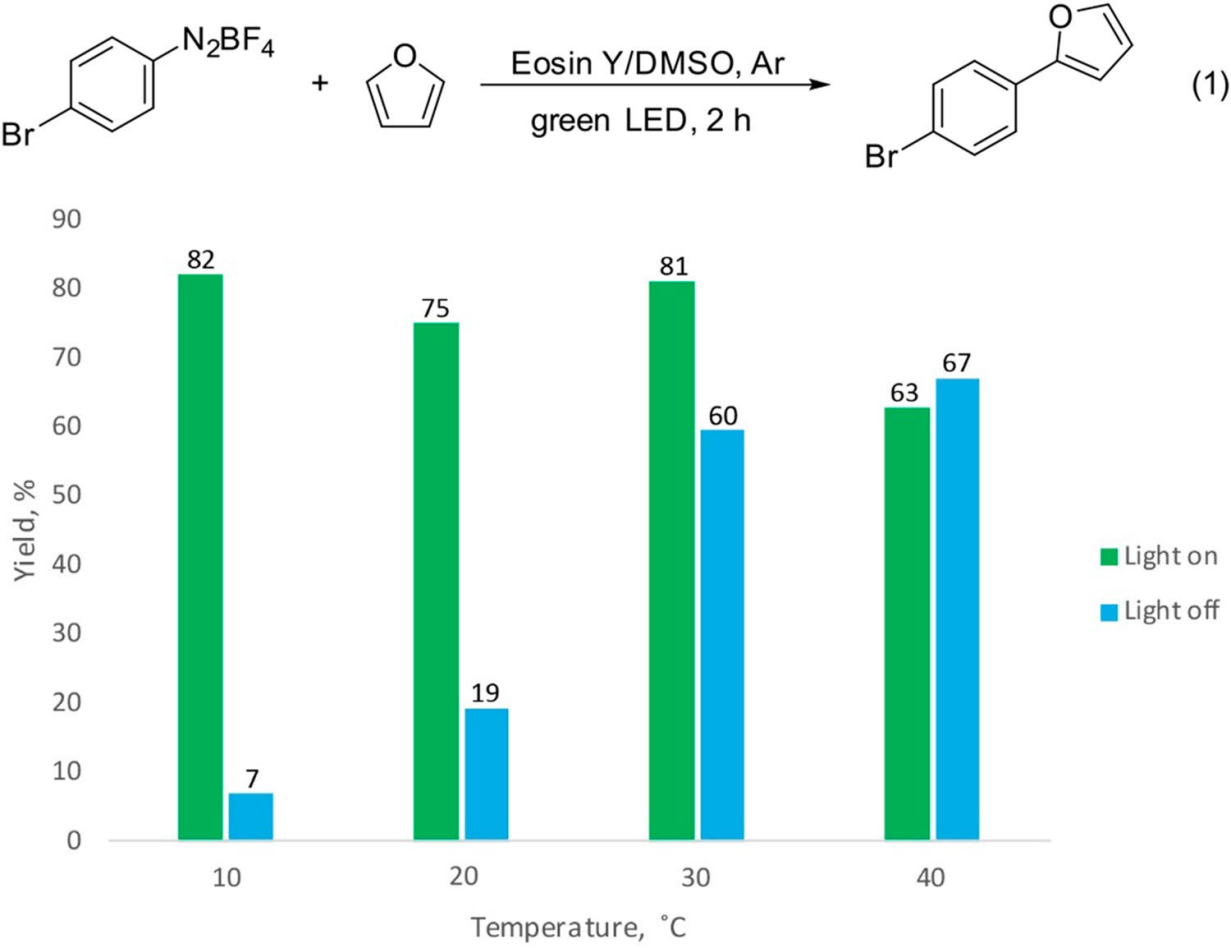
Model Eosin Y mediated arylation of furan. Yields were determined by ^1^H NMR.

To test the efficiency of the developed metal photoreactors, a model reaction of furan arylation at different temperatures was carried out (Fig. 3)^71–74^. Control experiments (without light) allowed us to analyze the dependence of the effect of photoredox catalysis on temperature. During the experiment, the reaction mixture was light-heated. If the temperature is noncontrolled, the photochemical effect vanishes to 25% at 30 °C and vanishes completely at 40 °C. With temperature stabilization at 10 °C, the photochemical route contributes to product formation in > 90%. The nonphotochemical route of the reaction can be associated with the thermal dediazoniation of the aryldiazonium salt promoted by DMSO^75^. Heating led to an increase in the rate of the nonphotochemical reaction. In other cases, heating of the reaction mixture by light irradiation or heat release may result in the formation of byproducts, leading to a lower yield of the target product, as well as to the need for additional purification. Thus, temperature control is a necessary condition for successful selective syntheses.

After optimization of the reaction conditions and development of the methodology of organic synthesis, the next step is to carry out the reaction on a large compound scale. To address this goal, we designed and built a custom-made device by FFF 3D printing. In contrast to the DMLS method, the FFF method is much cheaper and allows the production of large-volume reactors, which is well suited for solving problems of synthesis scaling-up. The development of an effective methodology for scaling up chemical synthesis is especially relevant in the pharmaceutical industry, since scaling can lead to an intensification and an increase in the number of side reactions due to a decrease in the efficiency of heat exchange and mixing of the reaction mass^76^. In the production of pharmaceuticals, especially high requirements are imposed on the purity of the obtained pharmaceutical drug; therefore, maintaining a high yield of the product and high selectivity of the chemical process is particularly desirable as a result of scaling up. Very often, in fine organic synthesis, scaling by simply increasing the reactor capacity is not effective, and it is necessary to develop a reactor of complex design to scale up the process while maintaining its efficiency. In this case, additive technologies are extremely effective since the ability to quickly manufacture complex components of chemical assembly is a key feature of 3D printing.

The layered quadratic design of a plastic photoreactor makes it possible to attach additional features, such as modules, for independent simultaneous addition of reagents into reaction vessels. Thus, the completely designed photoreactor consists of three module layers, each of which can be removed if necessary.

The first layer is a matrix of four 30 W LEDs (Fig. 2a). Each LED is separately attached to an aluminum cooling block with water, circulating through. The separation of matrices allows the easy replacement any of them by LEDs with different wavelengths. The support for LED blocks is printed with polycarbonate due to its stability at high temperatures. It reduces the damage in such cases as breakdown of the LED cooling system. Photoreactor consists of four 3D-printed syringe pumps, a programmed LCD display and a water bath connected to a thermostat (Fig. 2e). Measurement of light intensity directly in the reaction vessel provides more information for reproducing the experiment than the nominal power of LEDs. Thus, using the photodiode circuit, we measured irradiance in possible positions of reaction vials (directly under and between LEDs). The maximum power of light flowing through the reaction mixture in our setup is just 6% of the nominal 30 W of the LED matrix (Fig. 2d).

The second layer was designed for thermostating the reaction vessels (Fig. 2b). The use of gases and liquids as a thermostating medium allows us to carry out both scaling and optimization experiments. The high thermal conductivity of the medium minimizes the difference between temperatures inside and outside the reaction vessel. Thus, a water bath was chosen because of its higher thermal conductivity (0.598 W/m K at 20 °C) in comparison to air (0.026 W/m K at 20 °C)^77^. Polycarbonate was also chosen for printing the bath walls. The glass bottom, which should be transparent to visible light, was glued by epoxy. The simplest thermostat can be made by a typical heating plate with a PID controller and water pump connected with a bath through silicone tubes (Fig. 2b,e,f). A temperature stabilization experiment was carried out for a comparative study of the reactor (Fig. 2g). Thermal stabilization with a water bath provides high temperature stability and complete smoothing of temperature fluctuations, in contrast to a metal reactor.

Conventional plastic materials can operate in the temperature range up to 40–60 °C for polylactide (PLA), 60–70 °C for polyethylene terephthalate glycol (PETG), 90–110 °C for acrylonitrile butadiene styrene (ABS), 100–120 °C for polypropylene (PP), 140–160 °C for polycarbonate (PC). This temperature range (i.e., up to 100–160 °C) is usually enough for many organic transformations taking into account moderate boiling points of organic solvents. Nevertheless, some applications may require stability of the reactor at higher temperature. When using light sources with intense heating, substantial heating should be taken into account at the design stage of the reactor and sufficient additional cooling of the plastic reactor should be provided (for example, using a coolant supplied through multiple channels inside the reactor wall). Recently, thermoplastic materials for FDM printing characterized by a relatively high continuous operating temperature become widely available. For example, some materials based on polyamides provide continuous operating temperature up to 200 °C, while such materials are compatible with regular FFF 3D printing. Even more thermal stability up to 210 °C is possible with engineering polyether imide (PEI) plastics and up to 260 °C with polyether ether ketone (PEEK) plastic, which also compatible with 3D printing.

In photocatalytic processes, light sources with a high degree of heat release are replaced by LED-based sources, since unintended heating of the reaction mass can provoke side reactions and reduce the selectivity of the process.

Some reactions require gradual addition of a component (A) during the reaction time to keep its concentration at the required level (B). Optimization of conditions for such reactions can be accelerated by simultaneous usage of a few syringe pumps. Thus, based on open-source solution (Figs. S5, S6), we developed the third attachable module layer with four independently controlled syringe pumps (Fig. 2c,e). Linear, exponential or polynomial functions can be applied to control the rate of addition via software program code (see Supporting Information). The open-source concept provides an opportunity to program the syringe pump to fully control the dynamic system and achieve excellent yields.

**Figure 4:**
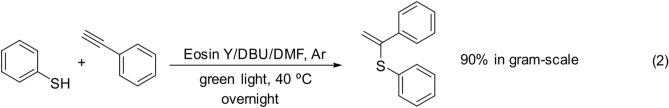
Studied Eosin Y-mediated photochemical thiol-yne coupling reaction.

To ensure that the 3D plastic printed photoreactor works properly, we provide a previously well-studied thiol-yne reaction^78^. The thiol-yne coupling between thiophenol and phenylacetylene provided 90% yield of the desired product after overnight. In this work, we scaled up the synthesis of vinylsulfide up to 10 times to a regular procedure. It is interesting to know that for the large-scale transformation, the desired vinyl sulfide was formed in excellent yield and selectivity (Fig. 4). Thus, the designed photoreactor has some obvious advantages: low cost, variable light sources, controllable rate of reagent addition, flexibility of different reaction vessel uses, upscaling and performing several reactions simultaneously.

## Conclusions

The present study explores two different types of 3D printing photoreactors: a type I reactor as a single temperature stabilization device for optimization of reaction conditions and carrying out many reactions in parallel (that result in saving time and accelerating discovery of new reactions) and a type II reactor for synthesis of important compounds on a larger scale.

The type I reactor has a compact body, which makes it possible to form arrays from them to accelerate the screening of chemical transformations at the same temperature, for example, when changing the radiation wavelength or the composition of the reaction mass. In addition, type I reactors can significantly reduce the cost of experiments because many individual temperature stabilization units multiply the cost of each experiment. Therefore, metallic reactors with rapid heat transfer and good stabilization in given experimental conditions are better suitable for fast optimization of the thermal regime. Despite the fact that metal reactors are manufactured by powder sintering, which may be characterized by microporosity in the final product, the thermal conductivity of DMLS reactors turned out to be quite comparable to the thermal conductivity of reactors made of compact metal.

The type II reactor of a much larger volume for the synthesis of large amounts of products is manufactured by the FFF method, which allows the manufacturing of large parts. This method is well suited for the manufacturing of reactors for scale-up organic synthesis. At the same time, despite the pronounced layered structure of the walls, which is formed by the FFF method, the reactor vessels turned out to be perfectly sealed. Taking into account the very wide range of thermoplastic materials suitable for FFF printing, including chemically resistant and heat-resistant materials, in our opinion, the use of plastics is preferable in all cases where high thermal conductivity or mechanical strength of metals is not required but dedicated temperature control for each reaction is desirable.

It is desirable to manufacture reactors for optimization of reaction conditions from metal due to its high thermal conductivity and short response time to temperature changes. Difficulties in the manufacturing of large reactors by the DMLS method may be associated with the need to optimize the parameters of additive manufacturing due to the possible significant shrinkage of the metal parts. While in the case of FFF printing, such optimization can be performed relatively easily, in the case of the DMLS method, this procedure requires high user qualifications.

A significant obstacle to the use of 3D metal printing is the high cost of equipment: the cost of an entry-level DMLS printer is much higher as compared to the plastic FFF 3D printer. However, metal 3D printing requires not only a printer, but also an expensive equipment for post-processing. Along with that, powder metals for 3D printing are in most cases more expensive as compared to plastics for FFF printing. Therefore, 3D metal printing for regular chemistry labs is often performed using third-party services that provide metal 3D printing services (which makes it affordable in price/accessibility terms). In contrast, FFF technology is easier available for direct laboratory use: an inexpensive 3D printer can be installed in the laboratory, and inexpensive and widely available consumables allow the production of many variants of reactors during the design optimization phase.

It is important to note that temperature control may be easily integrated into a 3D printed laboratory setup, which is extremely important since temperature control allows avoiding side processes in some cases, increasing the yield and selectivity of a chemical reaction, and changing the contribution of the photochemical effect to the overall process.

The present study points out the promising potential of 3D printing technologies in photochemical research. We anticipate rapid application of customized laboratory equipment in everyday laboratory practice in the near future. We supply 3D design files in standard STL format; thus, the designed reactors can be easily printed much faster than manufacturing with classical methods. This makes the overall procedure totally reproducible. The design can be further changed and adopted for other reactions. Additive manufacturing opens wide opportunities for creating new laboratory equipment with optimized functionality directly in the chemical laboratory in a short time.

## Methods

### 3D printing of the stainless steel reactor

The metal photoreactor was manufactured with an EOSINT M280 laser sintering system using a Yb-fiber laser to sinter metal powder. The part was made of PH1 stainless steel (layer thickness 20 μm), which is highly corrosion resistant. During additive manufacturing, metal supports were formed inside the central channel of the reactor, which were then removed mechanically.

### 3D printing of the plastic reactor

The PLA parts of the reactor were manufactured by FFF using a Picaso 3D Designer Pro 250 printer at a primary filament diameter of 1.75 mm. The PLA parts of the syringe pump were individually 3D printed with the parameters shown in Table 1. Additional 3D printed rafts and supports made of water-soluble polyvinyl acetate (PVA) were used for all PLA parts.

**Table 1:** Main parameters of FFF additive manufacturing for PLA and PC materials.

Part of the reactor	Material	Diameter of nozzle, mm	Temperature of build platform, °C	Extrusion temperature, °C	Cooling intensity, %	Extrusion multiplier	Layer height, mm
Syringe pump	PLA	0.3	60	220	40	0.90	0.20
Bath, LED holder	PC	0.5	100	265	0	0.95	0.40

The bath was printed using a Picaso Designer X desktop printer and polycarbonate filament with a 1.75 mm diameter. Additional 3D printed brim and special glue were applied for adhesion improvement (see Table 1 for additional printed parameters).

### Organic synthesis

Reaction (1): *p*-Bromophenyldiazonium tetrafluoroborate (0.115 mmol, 31.1 mg), Eosin Y (0.023 μmol) and furan (2.3 mmol, 0.167 ml) were dissolved in degassed DMSO (0.5 ml) in a 1.5 ml vial. The mixture was degassed and flushed with argon. Reactions were carried out at different temperatures for 2 h under 1 W green LED irradiation. Yields were determined by ^1^H NMR spectroscopy using trimethyl(phenyl)silane as an internal standard.

Reaction (2): 7 mmol of DBU and 0.03 mmol of Eosin Y were dissolved in 31 ml of DMF in a 250 ml flask to maximize the area of irradiation. The mixture was degassed under low pressure and filled with argon 3 times. After that, 2.6 mmol of phenylacetylene and 5.2 mmol of thiophenol were added to the mixture. The reaction was carried out overnight at 40 °C with four 30 W green LEDs.

## Supplementary Information

Supplementary Figures.

Supplementary Information.

## Data Availability

All data generated or analyzed during this study are included in this published article (and its Supplementary Information files).
