# Supplementary material for: Exploring metallic and plastic 3D printed photochemical reactors for customizing chemical synthesis
Source: Sci Rep. 2022 Mar 8;12:3780. doi: 10.1038/s41598-022-07583-9 (PMC8904794; doi:10.1038/s41598-022-07583-9)
Supplement: Supplementary file 1 — Supplementary Figures. [file 41598_2022_7583_MOESM1_ESM.pdf]

Supplementary Information

Exploring Metallic and Plastic 3D Printed Photochemical Reactors for Customizing Chemical Synthesis

Evgeniy G. Gordeev,[1] Kirill S. Erokhin,[1] Andrey D. Kobelev,[1,2] Julia V. Burykina,[1]
Pavel V. Novikov,[1] Valentine P. Ananikov[1,2*]

[1] *Zelinsky Institute of Organic Chemistry, Russian Academy of Sciences, Leninsky prospect 47, Moscow, 119991 Russia*

[2] *Lomonosov Moscow State University, Leninskie Gory GSP-1, 1-3, Moscow, 119991 Russia*

**val@ioc.ac.ru*

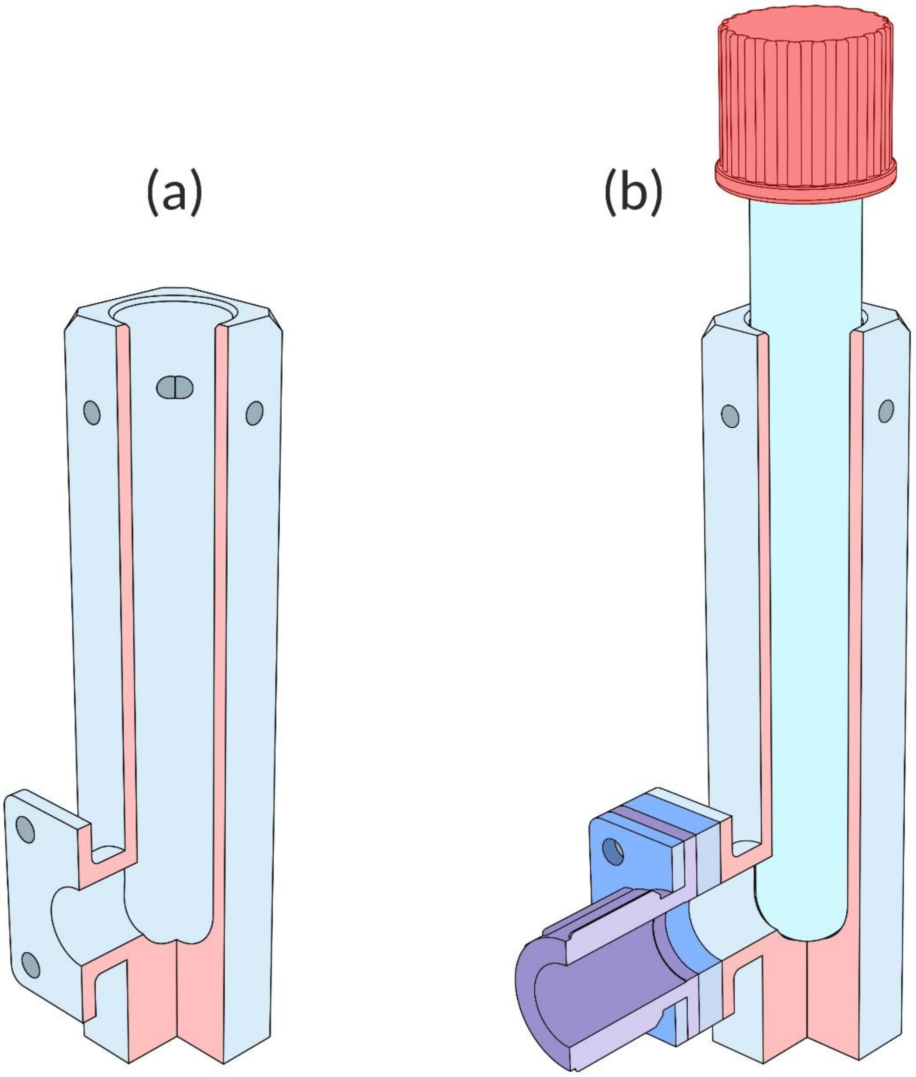

Figure S1. A sliced views of the photoreactor for optimization of the reaction conditions and manufacturing by DMLS: (a) without test-tube, (b) with inserted test-tube and LED mounting accessories.

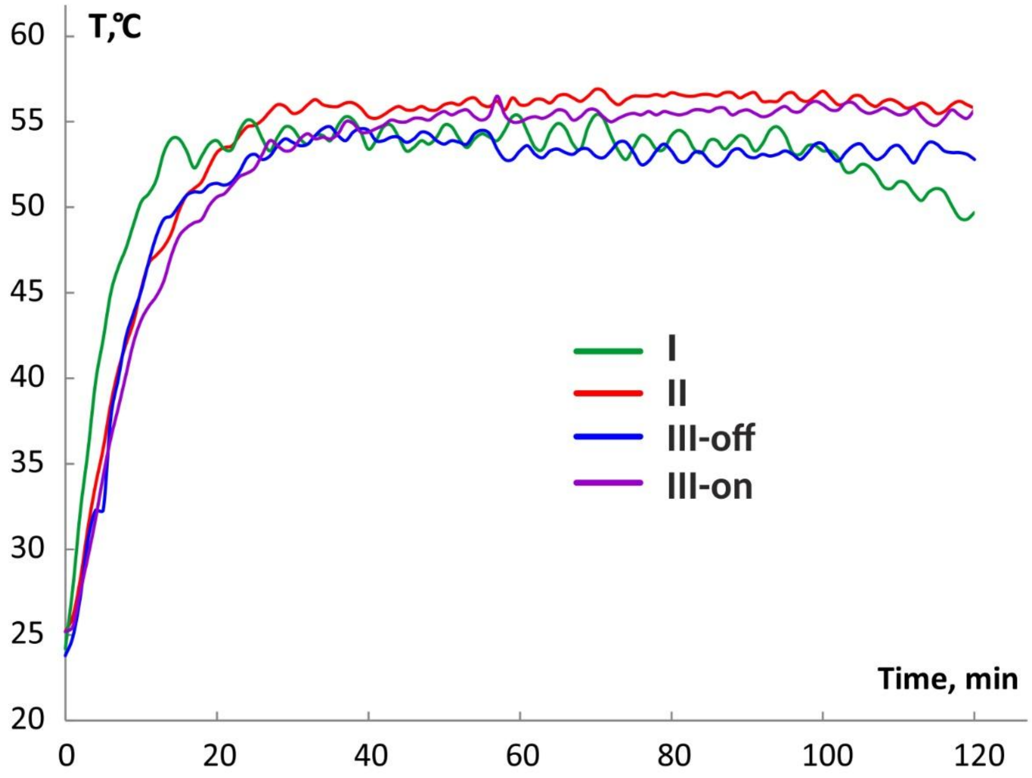

Figure S2. Monitoring of temperature stability over 2 hours in water at 50 °C for the following photoreactors: I – aluminum alloy and II – stainless steel reactors manufactured by conventional method; III-off and III-on - 3D printed stainless-steel reactor with LED switched off and with LED switched on, respectively.

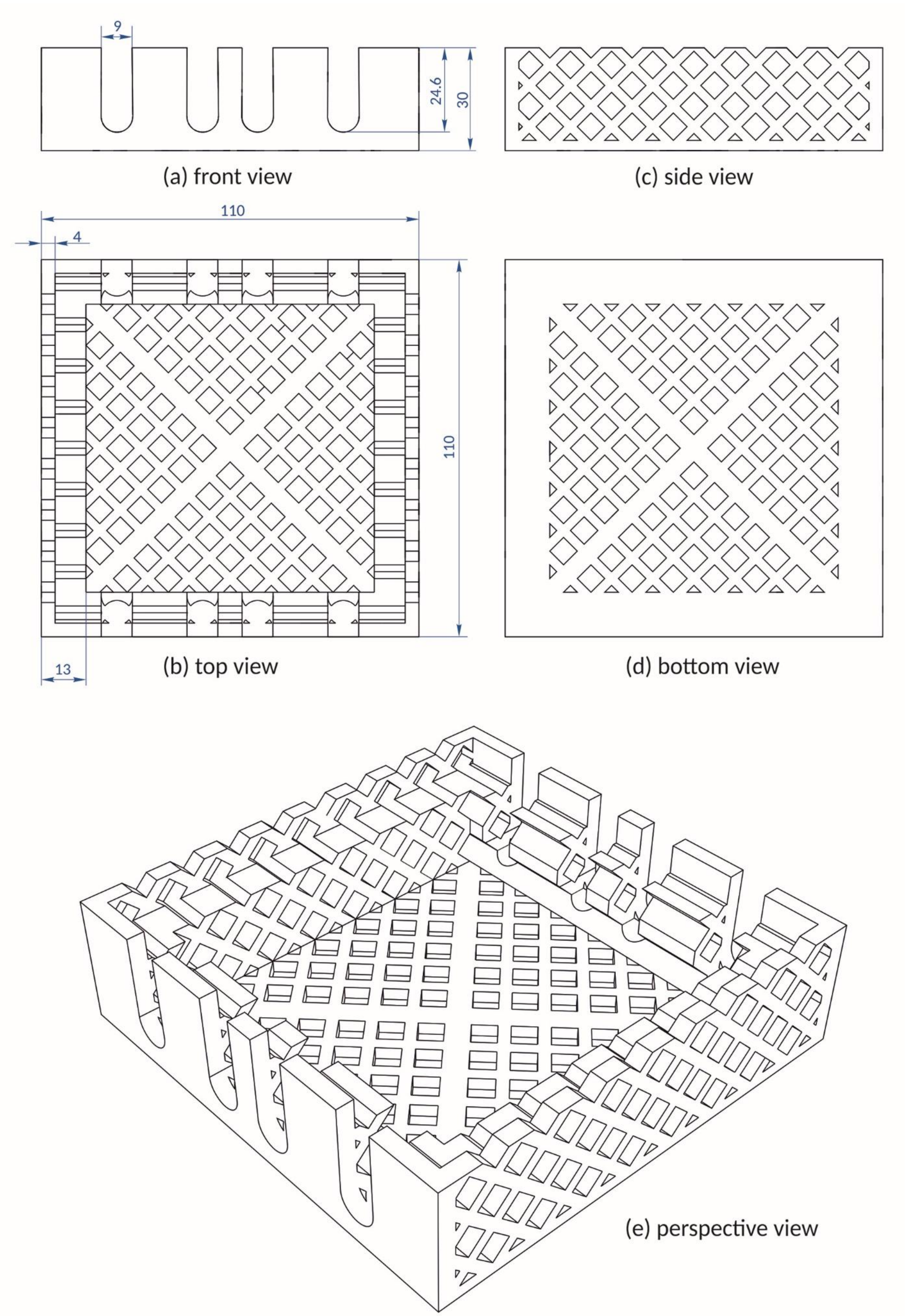

Figure S3. Mount box for LED array.

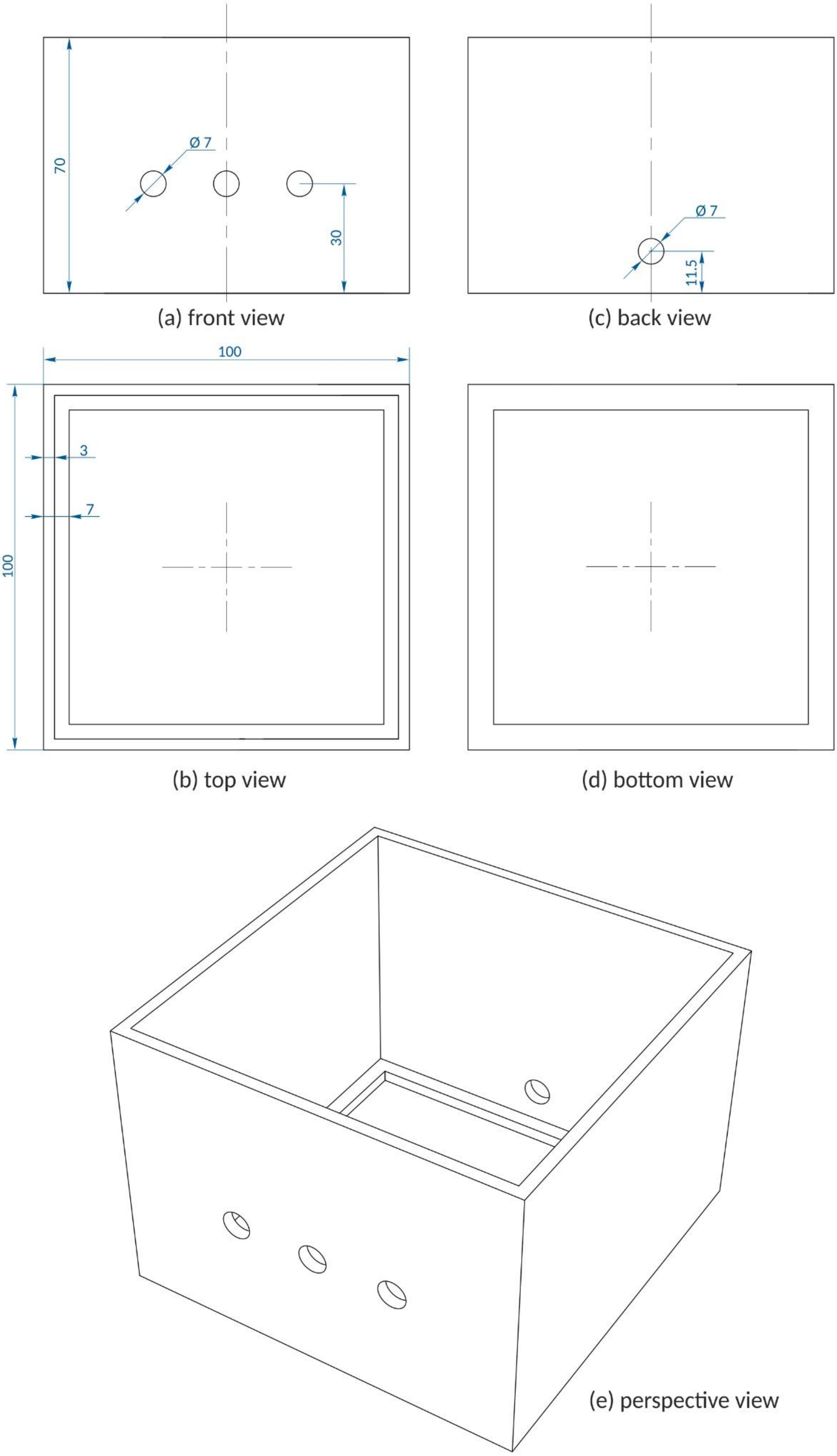

Figure S4. Full jacket with glass bottom for thermal stabilization of the reaction mixture.

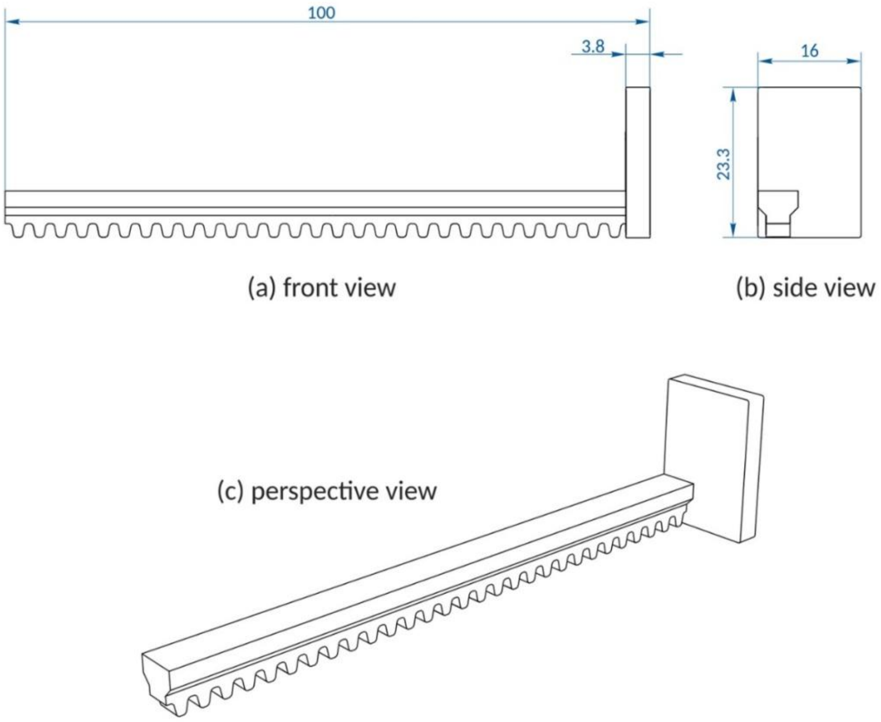

Figure S5. Linear actuator (pusher) for syringe pumps. Optimized version of open-source project https://www.myminifactory.com/object/3d-print-linear-servo-actuators-77542.

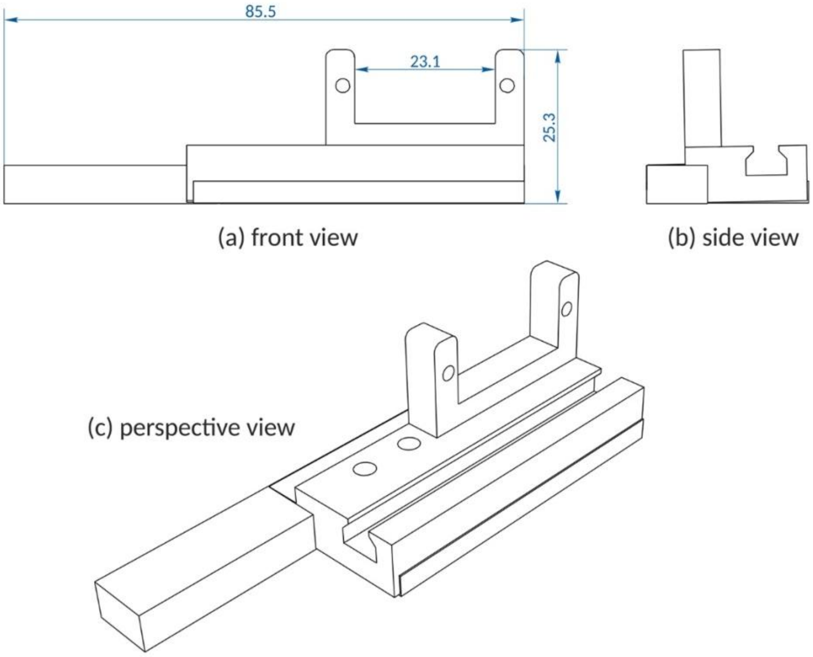

Figure S6. Stator for syringe pumps. Optimized version of open-source project https://www.myminifactory.com/object/3d-print-linear-servo-actuators-77542.

Additional material:

https://github.com/Ananikov-Lab/LightCube/tree/master/Models%20for%203d-printing%20of%20reactor%20parts

Code for programming syringe pumps can be found in GitHub repository https://github.com/Ananikov-Lab/LightCube
